# Entrapped Psychrotolerant Yeast Cells within Pine Sawdust for Low Temperature Wine Making: Impact on Wine Quality

**DOI:** 10.3390/microorganisms8050764

**Published:** 2020-05-20

**Authors:** Antonia Terpou, Vassilios Ganatsios, Maria Kanellaki, Athanasios A. Koutinas

**Affiliations:** Food Biotechnology Group, Section of Analytical Environmental and Applied Chemistry, Department of Chemistry, University of Patras, GR-26500 Patras, Greece; v.gkanatsios@gmail.com (V.G.); a.a.koutinas@upatras.gr (A.A.K.)

**Keywords:** sweet winemaking, psychrotolerant yeast, pine sawdust, freeze-drying, high-gravity low-temperature fermentation, aromatic profile.

## Abstract

An alternative methodology is proposed for low temperature winemaking using freeze-dried raw materials. Pine sawdust was delignified and the received porous cellulosic material was applied as immobilization carrier of the psychrotolerant yeast strain *Saccharomyces cerevisiae* AXAZ-1. The immobilization of yeast cells was examined and verified by scanning electron microscopy (SEM). The immobilized biocatalyst and high-gravity grape must were separately freeze-dried without cryoprotectants and stored at room temperature (20–22 °C) for 3 months. The effect of storage on the fermentation efficiency of the immobilized biocatalyst at low temperatures (1–10 °C), as well as on the aromatic characteristics of the produced wines was evaluated. Storage time had no significant effect on the fermentation efficiency of the biocatalyst resulting in most cases in high ethanol production 13.8–14.8% *v/v*. The volatile fraction of the produced wines was examined using headspace solid-phase microextraction (HS-SPME) followed by gas chromatography mass spectrometry (GC/MS). GC-MS/SPME analysis along with the organoleptic evaluation revealed in all produced wines a plethora of fresh and fruit aromatic notes. To conclude, fermentation kinetics and aromatic profile evaluation encourages the production of high-quality sweet wines at low temperatures using pine sawdust (*Pinus halepensis*) entrapped yeast cells as a promoter.

## 1. Introduction

Archaeometric studies date vine cultivation earlier and in regions of ancient Mesopotamia [1] while cultivation of grapes is known from Ancient Greece dating back at least 6000 years ago [2]. The technologies of viticulture and winemaking were widely developed along the Mediterranean region since grapes are an excellent raw material for winemaking. Nowadays, wine is an important component of the Mediterranean dietary tradition, while recent studies have moderated that its consumption reduces the incidences of coronary heart disease, atherosclerosis and platelet aggregation [3].

Towards a more sustainable processing of foods, the application of dry ingredients allows the industrial-scale production of standardized material and promotes the concept of global sourcing [4]. Drying of foods is a preservation method that aims to prolong the product shelf life and simplify its transport and storage. Freeze drying, or lyophilization, is a preservation process associated with food for minimum travel space, food for backpackers or ‘space food’. Freeze-drying yields a lightweight, high-quality, and easily rehydrated product that retains the original shape and most of the original constituents of the starting material [5]. As it has been demonstrated, bioactive compounds and vitamins, which are part of the grape raw material, remain unstable during storage [6]. On the other hand, the development of modern technologies for the preservation of food raw materials through freeze-drying allows the production of wine by freeze-dried grape must at any time of the year with lower costs [4,7].

Another important factor has been proved in recent years to be the valorization of agro-industrial waste attracting the attention of both industry and scientific research. Valorization of different residues has proved most intriguing exhibiting important results in many fields as is, for example, the valorization of lignocellulosic material which can be collected in large quantities from agricultural and forestry resources provided through upgrading biorefinery technologies fuels and chemicals [8]. Delignified cellulosic residues have also been applied as cell immobilization carriers being a low-cost high-abundant porous material deriving from many different agro-industrial applications [9,10]. For example, oak chips and cellulose powder have been successfully used for yeast immobilization in bottle-fermented sparkling wine [11] while wood sawdust has been successfully applied as a yeast cell immobilization carrier for alcoholic beverages production [12,13,14].

In recent decades new methods have been under study, targeting the improvement of the fermentation performance, aromatic profile and productivity of wines and alcoholic beverages [15,16]. It has been well established that the influence on the must composition and nutrients contained in must are of major importance regarding the evolution of the population of *Saccharomyces cerevisiae* in wine fermentation [17]. It is also well known that the aromatic profile of wine depends on various factors like grape variety, winemaking procedures and storage, maturation and aging, while the essential part of wine aroma is formed during alcoholic fermentation [18,19]. At the same manner, alcoholic fermentation at low temperature (4–20 °C) is a common practice used in white winemaking aiming to improve aromatic profiles by a high formation and retention of aromas. Furthermore, low temperature can improve the quality of wine in terms of volatiles, flavor, total acidity, pH, and alcohol production [14,20]. Another factor that has been proved to provide unique aromatic characteristics to wines is ageing in wooden barrels. As it is known from the ancient winery, when wood encounters wine valuable aromatic and flavor characteristics can be generated. This effect can be lost over time by the reuse of wooden barrels so alternative processes are currently being utilized by the wine industry such as the addition of wooden chips, sticks and staves [21]. Likewise, pine sawdust which is a cheap and abundant raw material may provide unique aromatic characteristics to wine products compatible to wooden barrel wine ageing [22,23].

The advantages of immobilized cell biocatalysts compared to free cell systems have been extensively revised through various natural supports, such as resins [15], fruits [24], membranes [25], gluten pellets [20,26] and delignified cellulosic residues [27,28,29]. Likewise, in recent years several immobilized yeast cell systems have been successfully proposed for use in alcoholic fermentation as yeast cells can be simply immobilized on a surface via natural adhesion [15,25,30].

Nowadays, making modifications to traditional practices or adopting novel bioprocess technologies has become of great interest in order to fulfill consumers expectations towards food products characterized by convenience, variety, adequate shelf-life, healthy properties, reasonable cost and environmental sustainability [15,31,32]. In this perspective, the role of emerging technologies in sweet winemaking is addressed towards enhanced volatile and flavor characteristics, reduced production time, optimized resources, extraction of high nutritional components provided by low temperature fermentation, high energy efficiency and extended shelf-life. Concerning the above-mentioned data and the need for novel, possible aromatic and cheap carrier systems of cell immobilization, the present work examines the use of *S. cerevisiae* AXAZ-1 psychrotolerant yeast immobilized on pine sawdust as a ready-to-use freeze-dried biocatalyst for low-temperature fermentation of high-density grape must. The main objective of current the study was to evaluate the efficiency of the produced biocatalyst along with high-density freeze-dried grape-must to perform alcoholic fermentation at low temperatures targeting the production of high-quality wine with enhanced aromatic characteristics.

## 2. Materials and Methods

### 2.1. The Psychrotolerant Yeast Strain S. cerevisiae AXAZ-1

The alcohol-resistant, psychrotolerant yeast strain *Saccharomyces cerevisiae* AXAZ-1 previously isolated from Greek grapes [33] was collected from the private collection of University of Patras. The yeast strain was grown in a synthetic medium consisting of (g L^−1^) 1 NH_4_SO_4_, 1 KH_2_PO_4_, 5 MgSO_4_ and 40 glucose previously sterilized (121 °C, 1–1.5 atm, 15 min). The sterile medium was inoculated with 4 g L^−1^ of the psychrotolerant yeast and incubated at 30 °C with aeration (500 cm^3^/min, 7 mbar) for 48 h. The culture was harvested by centrifugation at 5000 rpm for 10 min. For the cell immobilization procedure sterile synthetic media were prepared with the above composition of nutrients but containing enhanced glucose composition of 120 g L^−1^, pine sawdust and yeast suspension (see Section 2.2).

### 2.2. Tubular Cellulose as Immobilization Carrier

Pine sawdust was used as an immobilization carrier. Previously, pine sawdust was delignified by alkali treatment targeting increased porosity of the biocatalyst by lignin removal [12]. Delignified pine sawdust consisting mainly of cellulose (or tubular cellulose; abbrev TC) was autoclaved (121 °C, −1.5 atm, 15 min) prior use and used as carrier for the immobilization of the psychrotolerant and alcohol resistant yeast strain *S. cerevisiae* AXAZ-1.

The immobilization process was performed by suspending 7 g of the harvested wet yeast *S. cerevisiae* mixed with 20 g of dry tubular cellulose in 500 mL of glucose medium (120 g L^−1^) and leaving it undistributed at 30 °C for 24–48 h. When sugar content was below 0.1%, the fermented liquid was decanted, and the immobilized biocatalyst was washed twice with 12% (*w/v*) sterile glucose culture medium for free cells to be washed away. The immobilized cells were then frozen until −45 °C with a cooling rate of 3 °C/min. The frozen samples were freeze-dried under vacuum (5–15 × 10^−3^ bar) for 48–72 h in a Freeze Dry System, FreeZone 4.5 (Labconco, Kansas City, MO, USA). The condenser temperature was −44 ± 1 °C. The initial moisture content of the immobilized biocatalyst was 85–90% dry weight, and the dried material had a moisture content of approximately 5% dry weight. The immobilized biocatalyst was placed aseptically in air-tight containers with 14 g each and stored at 20–22 °C until use (0, 1, 2, 3 months).

### 2.3. Scanning Electron Microscopy

Freeze-dried pieces of pine sawdust along with freeze-dried pieces of the immobilized biocatalyst were coated with gold in a Balzers SCD 004 Sputter coater (Bal-Tec, Schalksmühle, Germany) for 2 min. The samples were examined in a JSM-6300 scanning electron microscope (JEOL, Tokyo, Japan), operated at an accelerating voltage of 20 kV. Scanning electron micrographs were obtained of both pine sawdust and the immobilized biocatalyst in order to reinsure yeast cell immobilization.

### 2.4. Preparation of Fermentation Composite

Grape must with an initial 12 °Be density and total acidity of 6 g tartaric acid/L, was frozen without cryoprotectants, until −45 °C (5 °C/min cooling rate). The frozen samples were freeze-dried under vacuum (5–15 × 10^−3^ bar) for 3–5 days with a condenser temperature at −45 ± 1 °C in a freeze-drying system as described above (Section 2.2). Finally, identical samples consisting of 68 g of lyophilized high-density grape must were prepared and stored at 20–22 °C to study the effect of storage time (0, 1, 2, 3 months).

### 2.5. High-Gravity Fermentation by Freeze-Dried Raw Materials

Winemaking with the freeze-dried raw materials included rehydration and mixing of the freeze-dried powders (grape must and immobilized biocatalyst). The freeze-dried immobilized biocatalyst was mixed with freeze-dried grape must and tap water was added to obtain the initial sugar concentrations. The freeze-dried powder mix was rehydrated with water in a ratio of 1:3 (1 kg of dry powder per 3 L of water) to a sugar content of 330 ± 2 g L^−1^ (glucose and fructose, 1:1). Specifically, 60 mL of tap water was mixed with 34 g of freeze-dried grape must reaching a total volume of 102 mL so that the initial sugar concentration was at the desired levels. The rehydrated grape-must was mixed with 14 g (dry mass) of the immobilized biocatalyst (containing 2.2 g of yeast representing a 11.9% of total volume) and introduced in a glass cylinder. This procedure was performed for all samples stored at various time intervals (30, 60 and 90 days, respectively) as well as for samples applied instantly for fermentation after preparation. Subsequently each mixture (immobilized biocatalyst and grape must) contained in 1 L cylindrical glass was placed for 48 h at 30 °C, and the temperature was gradually decreased to 10 °C within a period of 120 h and remained constant until the end of fermentation. The system was allowed to ferment in all cases without agitation. The fermentation was completed at 4.5–5° Be density as the fermentability of the biocatalyst was exhausted. The biocatalyst was collected using a sterile perforated fabric and the fermented liquid was filtered (1 micron beverage filter, BevBright, CA, USA) and stored at 5 °C [9]. No sulfur dioxide was added at the end of fermentation process.

### 2.6. Analytical Methods

#### 2.6.1. Sugar Analysis

Residual sugar was determined by high-performance liquid chromatography (HPLC), using Shimadzu chromatograph with a SCR-101N stainless steel column, a LC-9A pump, a CTO-10A oven at 60 °C and a RID-6A refractive index detector. Distilled water (3D) was used as mobile phase with a flow rate of 0.8 mL min^−1^ and 1-butanol was used as an internal standard. Samples of wine (0.5 mL) containing 2.5 mL of 1-butanol 1% (*v/v*) solution were diluted to 50 mL and 40 μL of the solution were injected into the column. The residual sugar concentration was calculated using standard curves and expressed as g of residual sugar/L.

#### 2.6.2. Ethanol Determination

Ethanol was determined on a Shimadzu GC-8A system, with a Teknokroma HAYE SEP Q 80/100 column, a C-R6A Chromatopack integrator, He as carrier gas with flow rate 20 mL/min, and a flame ionization detector (FID). The injection port and detector temperature were set at 210 °C. The column temperature was 130 °C. Wine samples of 2 μL were injected directly into the column. Determinations were performed by means of standard curves. Produced ethanol was expressed as % (*v/v*) of ethanol per total volume of substrate.

#### 2.6.3. Solid Phase Microextraction (SPME) Gas Chromatography/Mass Spectrometry (GC/MS) Analysis

The identification and semi-quantitative analysis of the headspace volatiles was conducted by means of gas chromatography/mass spectrometry (GC/MS) using the solid phase microextraction method (SPME). GC/MS analysis was performed on a Shimadzu GC-17A gas chromatograph coupled to a Shimadzu MS QP5050 mass spectrometer. The conditions of headspace SPME sampling were as follows: 10 mL of wine sample, 2 g of NaCl, and 1.6 mg/L internal standard (4-methyl-2-pentanol) were transferred into a 20 mL headspace vial fitted with a Teflon-lined septum sealed with an aluminum crimp seal. The sealed samples were stirred in a tap bath for 5 min at 60 °C in order to achieve the appropriate temperature, and then the fiber was exposed to the headspace for 30 min. The fiber used was a 2 cm fiber coated with 50/30 mm divinylbenzene/carboxen on poly(dimethylsiloxane) bonded to a flexible fused silica core (Supelco, Bellefonte, PA, USA). Desorption of wine volatiles took place in the injector of the gas chromatograph in the splitless mode, at 240 °C for 3 min. Helium was used as the carrier gas at a flow rate of 1.8 mL min^−1^. The volatile compounds separation was performed on a capillary column (Supelco CO Wax-10 60 m, 0.32 mm i.d., 0.25 μm film thickness). The oven temperature was programmed at 35 °C for 6 min and then it was raised to 60, 200, and 250 °C with a rate of 2, 5, and 25 °C min^−1^, respectively. Then the temperature was maintained at 250 °C for 6 min. The injector and interface temperatures were set at 240 °C and 240 °C, respectively. The mass spectrometer was operated in a scan range of 45–400 m/z. Molecular identification of volatile compounds was carried out by comparing the mass spectra obtained from NIST107, NIST21 and SZTERP libraries, and by determining Kovats retention indexes and comparing with those reported in the literature.

### 2.7. Sensory Evaluation by Principal Component Analysis

Sensory evaluation of sweet wines was determined using a panel of 10 previously trained assessors (5 males and 5 females from 25 to 60 years, median of 36.5 years) who were consumers of red wine and did not have allergies concerning its consumption. The evaluators were selected from the Laboratory of Food Biotechnology Group of Chemistry Department of Patras University as they were wine experts with wide experience in wine production and the capacity to recognize and describe wine aroma. For wine tasting, ISO standard wine glasses of 155 mm height and total volume of 215 mL were used, covered with Petri dish [34] filled with ~20 mL of wine at room temperature (~18 °C). Sensory analysis was carried out in panel booths conforming to international standards (International standard, 2007). Wine samples were presented to the panel in a random order identified by random three-digit codes [35]. The evaluators were asked to taste the samples and score the intensity of the following attributes: fruity, sour, fresh, bitter, alcoholic, sweet, and floral on a 0–10 cm unstructured linear scale (the higher the number the greater the intensity) anchored with the words ‘‘high intensity” and ‘‘absence” on the right and left ends, respectively [35]. Panelists evaluated each attribute using the ten-point scale to compare wines produced by the proposed technology according to storage effects (Section 2.4). Finally, overall acceptability of produced sweet wines was also assessed.

### 2.8. Statistical Analysis

A three-way repeated measures analysis was applied to the values of each volatile component and the results are presented as mean values ± standard deviation. The significance of differences in the means of various groups was checked by One-way Analysis of Variance (ANOVA) at the 5% level of significance. *p*-values below 0.05 were considered significant. Fermentations were carried out in duplicate using separately stored substrates. Principal component analysis (PCA) was used for identification of flavor and aroma attributes of produced wines and was computed using PanelCheck V.1.4.2.

## 3. Results and Discussion

### 3.1. Pine Sawdust Supported Biocatalyst Applied for Low Temperature Wine Making

Tree species of pine are considered to be the most common in the Mediterranean region. It is know that pine trees cover over 25,000 km^2^ of the Mediterranean and dominate the forest types in the semi-arid and dry regions [36]. Specifically, *Pinus halepensis* Miller (Alep’s pine), that was used in the present study, is one of the most important forest species in the Mediterranean region. As a result, pine sawdust is a highly abundant, low-cost material that can easily be prepared and handled.

**Figure 1:**
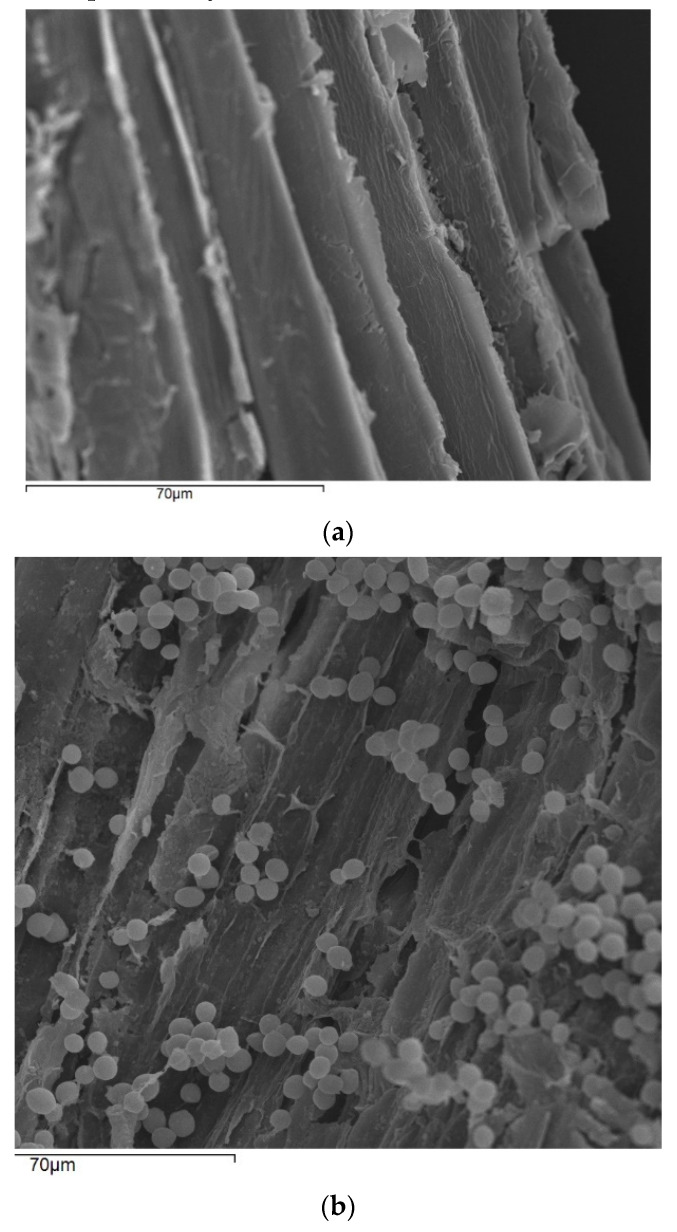
Scanning electron micrograph (×70 μm) of tubular cellulose origin from delignified pine sawdust (**a**), scanning electron micrograph (×70 μm) of delignified pine sawdust with immobilized *S. cerevisiae* AXAZ-1 cells (**b**).

**Figure 2:**
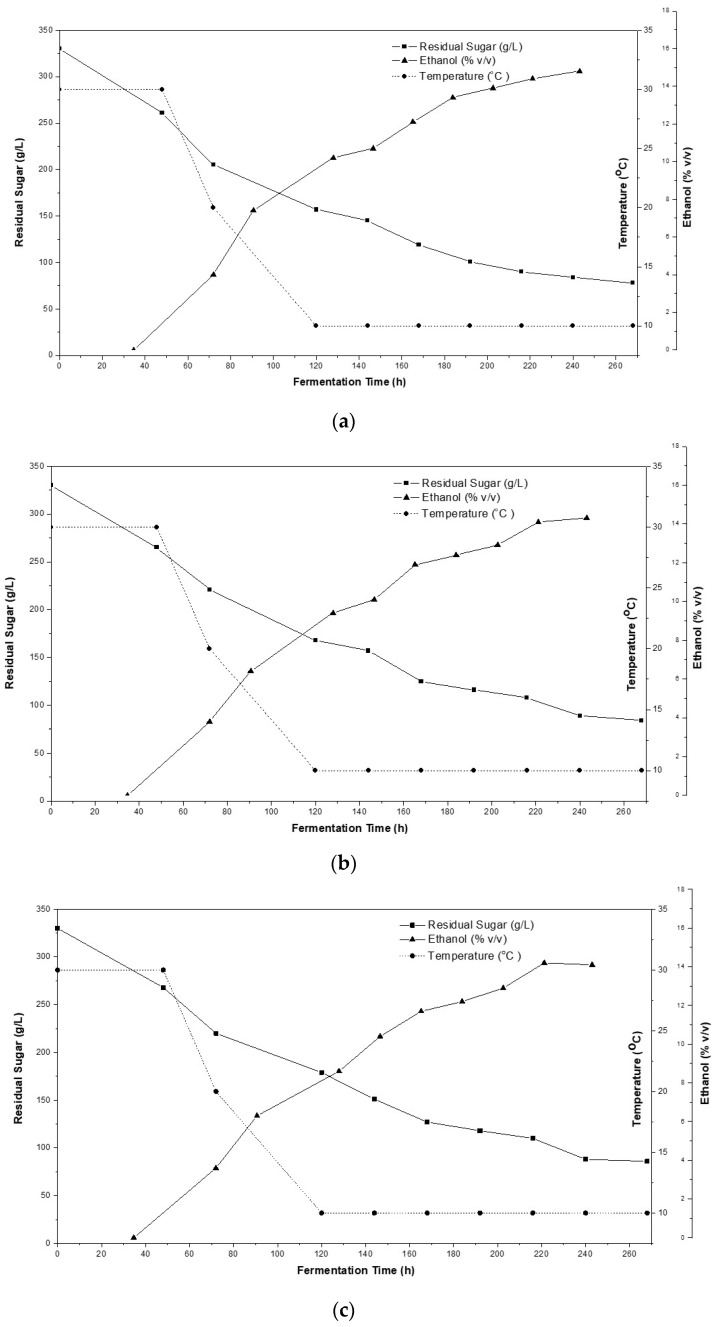

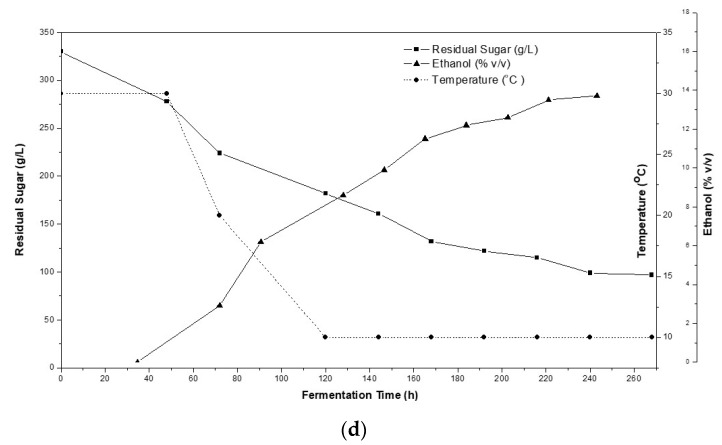
Kinetics of the sweet wine fermentation using freeze-dried raw materials after no storage time (**a**), storage for 30 days (**b**), storage for 60 days (**c**) and storage for 90 days (**d**) at room temperature (20–22 °C).

Tubular cellulose (TC), retrieved from pine sawdust after delignification, is a nano/micro-porous cellulosic material that has been proved to be suitable for use in food processing in support of immobilization [13]. In general, the multiple technological advantages offered by immobilized cells in alcoholic fermentations like improved fermentation productivity, cell stability, enhancement of cell viability and improved final product quality are well established [8,10,12]. In the present study the immobilized cells are attached on TC of delignified pine sawdust through physical adsorption by electrostatic and Van der Waals forces as well as by entrapment into the carrier tubes [12]. Cells adsorption on the support is a natural process archived as rough surfaces allows cell retention into the support’s cavities. The immobilization capacity of the biocatalyst was illustrated by electron microscopy (Figure 1), showing the delignified pine sawdust before and after immobilization, respectively. Likewise, the fermentation capacity of the immobilized biocatalyst is illustrated in Figure 2 showing sugar accumulation along with ethanol production as the biocatalyst was being stored in room temperature up to 90 days.

High gravity fermentations of freeze-dried grape must, which after rehydration obtained a sugar content of 330 g L^−1^, showed that the immobilized biocatalyst was capable to ferment the invert sugar even after 3 months of storage at room temperature (20 °C) without any significant effects on fermentation time and produced ethanol (Figure 2). Different studies have demonstrated that the osmotic effect caused by high sugar concentration, as provided in the present study (330 g L^−1^), can partially plasmolyze yeast cells, resulting in slow and/or incomplete fermentation [37,38,39]. Likewise, various others stress parameters which can negatively affect cell viability like metabolic imbalances and primarily resulting from bio-synthesis pathways [40]. On the other hand, as it has been proved by scientific studies that natural immobilization carriers can confer a protective effect to the cells against environment’s rough conditions [13,15,27]. Likewise, in the present study yeast cell immobilization along with an initiation temperature of 30 °C positively affected the fermentation process as the initial sugar content gradually decreased producing high amounts of ethanol (Figure 2). Moreover, as illustrated from fermentation kinetics the immobilized biocatalyst was able to perform high-gravity fermentation verifying previous studies of the multiple technological advantages offered by immobilized cells [8,10,12] as free psychrotolerant yeast strains may achieve alcoholic fermentation at low temperature [30] but they also need to provide good fermentation capacity and high tolerance when applied for wine making. Another important attribute is that TC presence might have increased the sugar uptake rate of the immobilized cells due to the attraction of hydrogen bonding onto the surface of TC [12,13].

In all cases the fermentation temperature gradually decreased from 30 °C down to 10 °C within a period of 120 h and the immobilized biocatalyst maintained its fermentability at low temperature producing sweet wines of high ethanol content. The initial high temperature was used to achieve a fast activation of the yeast starter culture [41] while low temperatures were applied targeting an enhanced aromatic profile of produced wines [42]. Specifically, by applying gradual fermentation temperature from 30 °C down to 10 °C, an alcoholic liquid interlaced with an alcoholic content in the range of 14.8–13.8% *v/v* and sugar content 78–97 g L^−1^, respectively (Figure 2). Storage time of the biocatalyst at room temperature significantly affected the ethanol production of the produced wines. Specifically, there was observed significantly lower ethanol production in wines produced by freeze-dried raw material after 90 days of storage compared to wines produced after instant production of freeze-dried raw materials. This result was expected as nutrients contained in grape-must may be altered during storage even within a freeze-dried form and as a result, yeast shows a lower fermentation capacity when important nutrients are lacking from the fermentation medium. Another important factor which can affect the fermentation capacity and ethanol production of the biocatalyst is the viability of yeast cells. During storage, especially at temperatures higher that 4 °C, a significant amount of viable cells may be lost [43]. Nevertheless, in the current study it is highlighted that optimum ethanol rates up to 14% *v/v* can be achieved by proposed freeze-dried mixture even after to 2 months of storage at room temperature (20–22 °C). For all cases, sweet wine of good quality was retrieved after fermentation of approximately 11 days.

The present procedure was proved to be effective for the preparation of sweet wine, without addition of potable alcohol. The use of immobilized cells on delignified pine cellulose contributes to: (i) successful fermentation at low temperatures (Figure 2) and (ii) extraction of resins aromatic compounds into the fermented wine (Table 1).

### 3.2. Effect of Storage on Wines Aromatic Characteristics

Semi-quantitative analysis of volatile compounds was performed by SPME GC/MS targeting to evaluate the effect of freeze-dried raw materials storage time (0, 30, 60 and 90 days) on the aromatic characteristics of produced wines (Table 1). The majority of identified compounds were esters that show low thresholds values and are considered to confer a major impact on wine flavour and aroma. In addition, various organic acids, carbonyl compounds, alcohols and terpenoids were also determined in all wine samples.

In general, most compounds that contribute to the aroma of wines are produced during must fermentation while very few of them can be derived from grapes. In our case it is possible that aromatic compounds could also be retrieved from pine sawdust [36]. This factor is assumed since *Pinus halepensis* trees, where pine sawdust was retrieved, is used to produce the famous Greek wine called “retsina” by addition of the resin in the must during fermentation [44]. Retsina is a white or rose wine known from the ancient times. The resin which is derived from the *Pinus halepensis* tree has a major aromatic impact on ‘retsina’ wine while it can also act as a preservative [44,45]. Likewise, pine sawdust might include the resins aromatic characteristics which could attribute to the aromatic profile of produced wines (Table 1).

Terpenes are typical varietal aroma compounds present in grapes in free volatile forms and bound with sugar as glycosides. Free terpenes have low aroma thresholds, so they have a high impact on “floral” character of wines. Their concentrations in grapes are influenced by several factors like maturation degree, soil conditions, viticulture practices and grape cultivar [42]. In produced wines terpenes are most likely deriving from the immobilized biocatalyst and specifically the pine sawdust applied as support of immobilization as well as the grape must. Specifically, terpenes contained in grapes can also be synthesized during fermentation after hydrolyzation of glyosidic combinations [54,55] and add a flowery sensory note to wine aroma. Some terpenes (anethol, geranyl acetone, nerolidol, farnesol) were detected in wines produced by freeze-dried raw material applied for fermentation instantly after production while they were not detected in wines prepped with freeze-dried raw material after longer periods of storage. It is most likely that these terpene compounds were affected by storage conditions. Concerning some other terpenes like linalool, α-terpineol and β-citronellol, it was observed that they could provide their floral characteristics equivalently in all produced wines. As a result, storage of the freeze-dried raw material did not have an impact on these aromatic terpenes.

It has been documented that there is a positive correlation of wine quality with esters content (within certain limits) [56]. The majority of esters are produced during fermentation as a result of yeast metabolism [57]. Ethyl esters of fatty acids as well as acetates of higher alcohols are associated with a flowery, fruity aroma in the products and are highly desirable in wines. In most cases, during 90 days of storage, esters concentrations were generally decreased. Additionally, there are other volatile compounds formed during yeast fermentation like higher alcohols, medium chain fatty acids and their corresponding esters, which also play an important role in the overall aroma of the young white wine. All these compounds can be affected by several fermentation factors like storage time and fermentation temperature.

The alcohols detected in wines are mainly aliphatic, amyl or aromatic fusel alcohols. If the concentration of these alcohols is detected in levels below 300 mg/L they intensify floral notes in wines, whereas they are detrimental to wine quality when their concentration surpasses that threshold [58]. Phenyl- ethyl alcohol may provide sweet rose-like flavours that positively contributes to sweet wine aroma even at low concentrations [59,60]. In our case, phenyl-ethyl alcohol was detected in the sample obtained from the 90-days stored raw materials. The number and the concentration of esters was detected higher, compared with the group of alcohols (Table 1).

Concerning acids detected in wine samples, octanoic and n-decanoic acid were observed in all the samples regardless the storage time of the raw freeze-dried materials. Below threshold levels, they contribute to the complexity of the aroma bouquet, but above threshold values they have a negative [61]. Shinohara et al. reported that the presence of C_6_-C_10_ acids in wines at 4–10 mg L^−1^ concentrations provided a pleasant aroma, but at concentrations higher than 20 mg L^−1^ had a negative effect [62]. The concentrations of those acids in our study were detected low compared to all other aromatic by-products contributing to the high quality of the produced wines.

In general, volatile evaluation highlights the good quality of produced wines indicating the positive influence of the immobilized biocatalyst in the aromatic profile of produced wines.

### 3.3. Discrimination of Wine Flavor and Aroma Attributes

**Figure 3:**
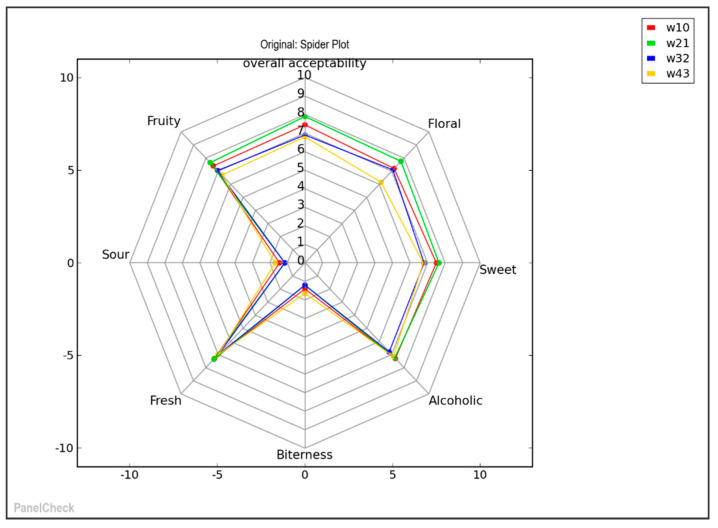
Wines sensory evaluation presented as a spider plot. The research wine samples were prepared by the proposed technology of freeze-dried raw materials without prior storage (W10) and after storage for 30 (W21), 60 (W32) and 90 (W43) days, respectively.

All wines produced by the proposed technology achieved high scores of acceptability (illustrated by wines’ sensory spider plot; Figure 3) and were characterized by harmonious taste and floral, fresh fruity notes. As highlighted by the aromatic profile evaluation determined by GCMS/SPME in the previous section, the most abundant compounds detected in wines were esters, higher alcohols, and volatile fatty acids. These compounds are considered as important contributors to the fermentation bouquet of wine and are in agreement with results retrieved by sensory evaluation verifying the floral, fruit and fresh character of produced wines (Figure 3). Regarding overall acceptability the evaluation panel showed a higher preference towards wines produced by freeze-dried raw material with no storage (W10) and 1 month of storage (W21), respectively. The fresh attribute of produced wines showed no significant differences resulting to the optimum quality of all produced wines. Meanwhile, some differences were detected regarding the floral attributes of wines with the ones produced by stored freeze-dried raw material testing the lowest scores. This result is in accordance with GCMS analysis showing terpene and ester content in wines produced with freeze-dried raw material stored for a longer period. 

**Figure 4:**
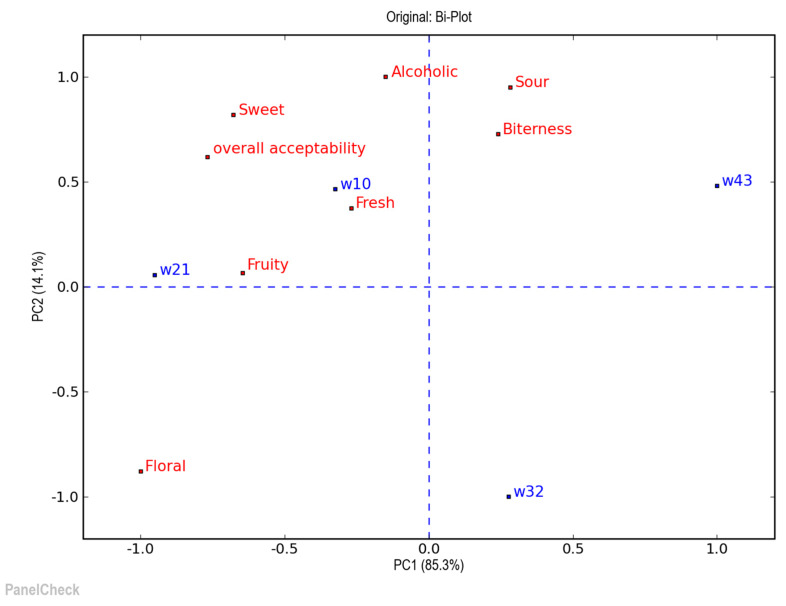
Wine sensory attributes presented by graph of principal component analysis (PCA). The research wine samples were prepared by the proposed technology of freeze-dried raw materials without prior storage (W10) and after storage for 30 (W21), 60 (W32) and 90 (W43) days, respectively.

The results of principal component analysis concerning flavor and aroma attributes of produced wine samples are summarized in Figure 4. The most important characteristic of wine is aroma [63]. According to the evaluation panel wines W10 and W21, which cluster together, were characterized by fresh and fruity aromatic characteristics. Panelists noted an enhanced bitterness in wine W43, while wine W32 was characterized as sweeter compared to the other samples. This result could be expected in wines W32 and W43 where the final sugar content was higher compared to other wines. Most likely this attribute was not recognized by evaluators in wine sample W43 due to the enhanced bitterness. In general, higher sugar content was retrieved in samples W32 and W43, most likely due to the higher storage time of the immobilized biocatalyst which might have affected its fermentation capacity.

## 4. Conclusions

Winemaking comprises a diverse set of factors that play a crucial role during the transformation of grapes to wine [19]. The most important factors generally considered by winemakers include vineyard management, grape quality, winemaking practices, and the proper use of commercial selected yeasts. Increasing consumer demand for good quality wine in addition with the emerging demand for highest added value products has led to the manufacture of a novel marketable dried mixture for high-quality wine making at low temperature. A new procedure of freeze-dried raw materials containing immobilized psychrotolerant yeast cells on pine sawdust (low cost cellulosic residues) and high-density grape must for low temperature winemaking has been developed. All produced wines were free of preservatives and the freeze-dried raw materials maintained their fermentability up to 90 days of storage providing high quality aromatic wines at a low temperature of approximately 14% *v/v* alcohol. Pine sawdust proved suitable as support for yeast cell immobilization, possibly providing in parallel some aromatic characteristics of low threshold value to the produced wines. As a result, this novel procedure has been demonstrated as successful and can be tested in the near future for industrial applications.

## Figures and Tables

**Table 1:** Volatile compounds (μg/L) identified by solid phase microextraction and gas chromatography/mass spectrometry (SPME GC/MS) technique in wines produced at low temperature using stored (0–90 days) freeze-dried raw materials (grape must and immobilized *Saccharomyces cerevisiae* AXAZ-1 yeast cells on pine sawdust).

Identified Compound	ID **	KI **	KI_ref_ **	Content of Volatile Compounds in Samples (μg/L)			
				Wine 1 *	Wine 2 *	Wine 3 *	Wine 4 *
***Esters***							
Ethyl acetate	MS, KI	886	894 ^a^	3692.8	3934.7	2389.7	940.9
Ethyl butanoate	MS, KI	1018	1040 ^e^	244.9	298.03	145.1	74.7
Ethyl 2-methyl butanoate	MS, KI	1036	1063 ^c^	234.5	180.5	200.6	190.6
Ethyl pentanoate	MS, KI	1054	1148 ^f^	110.3	125.6	95.4	88.5
Isoamyl acetate	MS, KI	1109	1123 ^e^	654.5	1566.3	390.1	745.7
Ethyl hexanoate	MS, KI	1212	1250 ^f^	2965.7	4809.6	9668.7	9887.1
Hexyl acetate	MS, KI	1253	1281 ^c^	334.2	255.9	613.12	867.5
Ethyl octanoate	MS, KI	1386	1421 ^f^	7980.6	11,359.3	4626.1	2257.7
Ethyl decanoate	MS, KI	1599	1635 ^e^	2696.2	5248.9	3417.1	4138.7
Diethyl succinate	MS, KI	1639	1679 ^h^	6882.7	6304.2	6072.6	4229.8
Ethyl 9-decenoate	MS, KI	1659	1692 ^h^	875.6	613.1	1088.5	1584.2
Ethyl benzeneacetate	MS, KI	1762	1809 ^e^	87.2	88.5	70.6	73.1
2-Phenylethyl acetate	MS, KI	1796	1832 ^a^	884.1	2723.3	3489.2	2277.8
Ethyl dodecanoate	MS, KI	1821	1850 ^e^	133.8	102.4	164.5	197.8
2-Phenylethyl butanoate	MS, KI	1954	1930 ^c^	304.2	337.9	287.1	231.4
Isopropyl myristate	MS, KI	2030	2041 ^e^	1204.0	2613.0	735.19	441.44
Ethyl tetradecanoate	MS, KI	2036	2056 ^c^	250.5	230.7	200.8	220.4
***Alcohols***							
4-methyl 1-pentenol	MS, KI	1253	1282 ^e^	39.5	40.2	28.7	29.4
3-penten-1-ol	MS, KI	1310	1305 ^d^	184.5	194.1	170.5	105.6
1-Hexanol	MS, KI	1331	1340 ^b^	590.0	1059.8	1093.4	745.5
3-Methyl 1-pentanol	MS, KI	1353	1350 ^e^	131.5	122.5	130.5	110.4
2-ethyl 1-hexanol	MS, KI	1445	1490 ^d^	2015.6	1805.4	1742.3	1889.7
1-Heptanol	MS, KI	1463	1464 ^a^	112.3	144.2	115.7	110.8
1-Octanol	MS, KI	1512	1536 ^f^	112.4	111.9	496.0	518.09
1,3- Butanediol	MS, KI	1616	1590 ^e^	859.5	nd	nd	nd
Phenylethyl alcohol	MS, KI	1896	1938 ^a^	11,487.2	10,254.3	3078.2	1794.7
***Acids***							
Hexanoic acid	MS, KI	1873	1851 ^f^	846	nd	nd	nd
Octanoic acid	MS, KI	2078	2111 ^a^	11,671.12	12,877.61	1257.12	2704.15
n-Decanoic acid	MS, KI	2293	2336 ^e^	6125.86	7235.31	5323.95	3199.69
***Carbonyl compounds***							
Nonanal	MS, KI	1349	1395 ^f^	33.5	nd	35.2	40.5
Furfural	MS, KI	1426	1475 ^a^	350.6	360.2	343.5	393.3
Decanal	MS, KI	1457	1507 ^a^	190.2	nd	nd	nd
***Terpenoid compounds***							
D-Limonene	MS, KI	1119	1189 ^b^	241.2	180.5	155.6	124.7
Linalool	MS, KI	1500	1500 ^i^	775.9	2369.2	1268.6	392.9
α-Terpineol	MS, KI	1665	1661 ^n^	850.5	976.5	737.2	700.6
β-Citronellol	MS, KI	1733	1711 ^h^	112.3	111.3	110.3	110.5
Anethole	MS, KI	1819	1843 ^e^	156.1	nd	nd	nd
Geranyl acetone	MS, KI	1841	1853 ^g^	663.6	nd	nd	nd
Nerolidol	MS, KI	2030	2053 ^c^	316.8	nd	nd	nd
Farnesol	MS, KI	2283	2343 ^e^	5277.6	4512.6	4156.3	4215.2

* Wine 1, 2, 3, 4: wines produced without storage of raw materials and after storage of 0, 30, 60 and 90 days, respectively. ** ID: Method of identification, KI: tentative identification by Kovats retention index, KI_ref_: tentative identification by Kovats retention index in accordance to the literature (^a^: Riu-Aumatell, et al. [46],^b^: Schoina, Terpou, Bosnea, Kanellaki and Nigam [35],^c^: Wong and Teng [47], ^d^: Kandylis, et al. [48], ^e^:Kandylis, et al. [49], ^f^: Terpou, et al. [50], ^g^: Riu-Aumatell, et al. [51], ^h^:Kandylis, et al. [52], ^i^: Smadja, et al. [53]). nd: not detected on this column.
